# Robot-Assisted Laparoscopic Adrenalectomy for Rare Myxoid Adrenocortical Carcinoma

**DOI:** 10.1155/2019/9794345

**Published:** 2019-12-21

**Authors:** Grant Johnson, Kiran Thalody, Umesh Kapur, Thai T. Nguyen

**Affiliations:** ^1^Franciscan Health Olympia Fields Hospital, Midwestern University, Downers Grove, IL, USA; ^2^ROWAN SOM, Stratford, NJ, USA; ^3^Comprehensive Pathology Services, Silvercross Hospital, New Lenox, IL, USA; ^4^Advanced Urology Associates, Silvercross Hospital, New Lenox, IL, USA

## Abstract

**Background:**

Surgical resection remains the standard treatment for adrenocortical carcinoma. Higher rates of local and peritoneal recurrence have been reported with the laparoscopic approach compared to open resection, although the evidence is limited. A dilemma occurs when tumors appear benign in nature, measure >5 cm, or when patients request a minimally invasive surgical approach. We describe the first reported case to date of successful robot-assisted laparoscopic adrenalectomy for myxoid variant adrenocortical carcinoma.

**Case Presentation:**

A 38 year old female presented with a large 8.0 cm enhancing left adrenal mass concerning for pheochromocytoma, given refractory hypertension and symptoms of palpitations and headaches. Functional work up was negative. The patient underwent robot-assisted laparoscopic left adrenalectomy after appropriate alpha and beta blockade. Histological sections demonstrated a cortical neoplasm with prominent myxoid changes consistent with myxoid adrenocortical carcinoma. The patient's symptoms resolved and serial imaging demonstrated no recurrence three and nine months, post-operatively.

**Conclusion:**

Myxoid adrenocortical carcinoma is a rare and aggressive entity best managed with surgical resection. Though open resection for invasive adrenal cancer remains the gold standard, minimally invasive approaches are being increasingly used, whether deliberately or not. We describe the first reported robot-assisted laparoscopic adrenalectomy for invasive myxoid adrenocortical carcinoma.

## 1. Introduction

Surgical resection remains the standard treatment for adrenocortical carcinoma (ACC). Open adrenalectomy has historically been used for large invasive tumors concerning for ACC, but laparoscopic adrenalectomy has been used for smaller lesions given potential advantages of decreased post-operative pain, shorter hospital stay, and faster recovery. Higher rates of local and peritoneal recurrence have been reported with the laparoscopic approach compared to open resection, although the evidence is limited. A dilemma occurs when tumors appear benign in nature, measure >5 cm, or when patients request a minimally invasive surgical approach. We describe the first reported case-to-date of successful robot-assisted laparoscopic adrenalectomy for myxoid variant ACC, a rare tumor of which only 42 cases have been reported worldwide.

## 2. Clinical History

A 38 year old female presented with a history of hypertension refractory to medical therapy. Given concerns for renovascular hypertension, she underwent abdominal ultrasound demonstrating a left adrenal mass. She was referred to urology for further work up and management. The patient denied any associated flank or abdominal pain, however, she did report experiencing palpitations and headaches with elevated blood pressure measurements. She denied diaphoresis, flushing, chest pressure, or visual disturbances. The patient denied any family history of Multiple Endocrine Neoplasia Syndrome (MEN) disorders, pheochromocytoma, or markedly elevated blood pressure. She had no personal history of diabetes or recent symptoms to suggest Cushing's syndrome. She denied any family history of adrenocortical carcinoma.

## 3. Physical Exam

She was noted to have a blood pressure of 170/100 mmHg without tachycardia. Body mass index was 36.8 kg/m^2^. General appearance was normal without evidence of truncal obesity, moon facies, nuchal fat pad. Skin exam did not reveal violaceous striae or facial flushing. Cardiac, lung and abdominal exams were unremarkable without palpable mass or CVA tenderness. Neurologic and musculoskeletal exams were normal.

Laboratory evaluation demonstrated normal serum electrolytes, including normal serum potassium. She underwent work up for metabolic function of the adrenal lesion with normal serum catecholamines (norepinephrine 283 ng/mL, epinephrine <15 ng/mL, dopamine <30 ng/mL) and normal 24 urine collection for free serum cortisol (10.9 mcg/24 hr, 24 urine volume 1675 mL).

**Figure 1:**
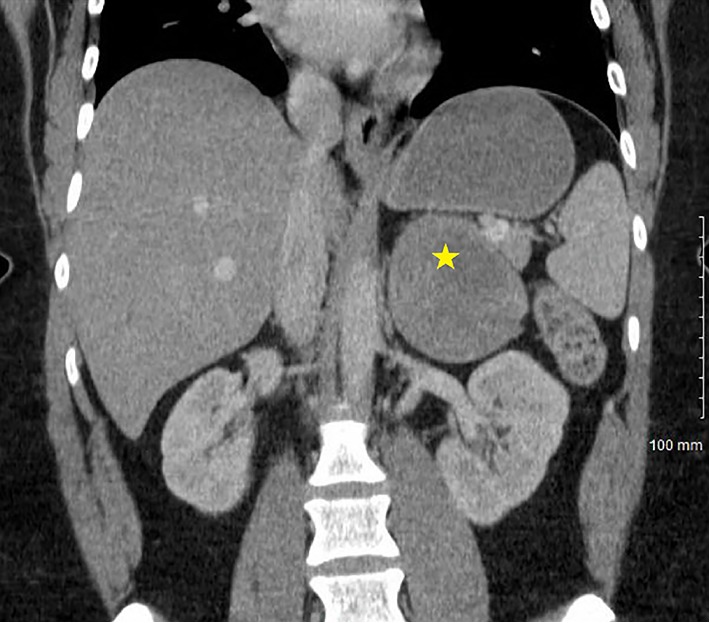
Contrast enhanced computed tomography imaging of abdomen, coronal view: left adrenal mass measuring 7.4 × 7.3 × 8.0 cm (star).

Imaging work up was performed including computed tomography (CT) scan with intravenous contrast revealing a large enhancing left adrenal mass measuring 7.4 × 7.3 × 8.0 cm with no washout on delayed imaging (Figure 1). Magnetic resonance imaging (MRI) of the abdomen with and without contrast, using Gadolinium (Gabobutrol) demonstrated a 7.4 × 7.4 × 8.0 cm heterogeneously T2 hyperintense and heterogeneously enhancing LEFT adrenal mass, highly suspicious for pheochromocytoma.

## 4. Diagnosis

Left adrenal mass measuring 8.0 cm in greatest dimension, suspected pheochromocytoma based on MRI characteristics, with negative functional work up. No biopsy was performed given underlying concern for pheochromocytoma.

## 5. Intervention

**Figure 2:**
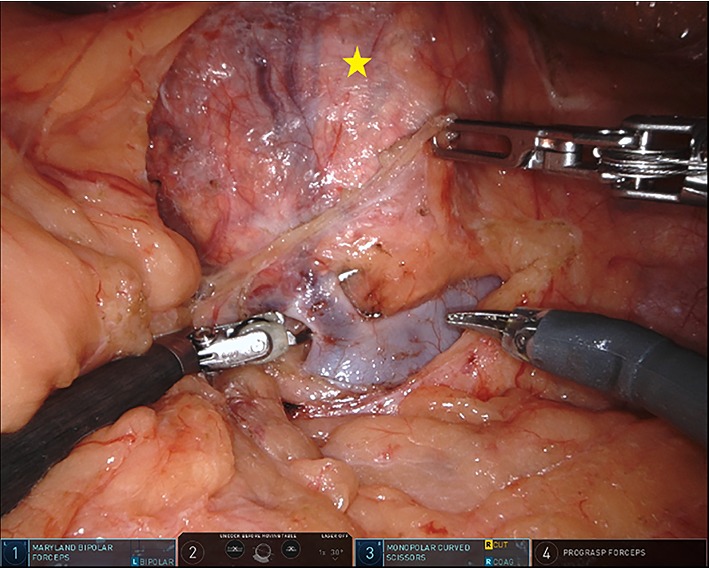
Left adrenal tumor (star) with exposed left renal and suprarenal vein during robot-assisted laparoscopic adrenalectomy.

**Figure 3:**
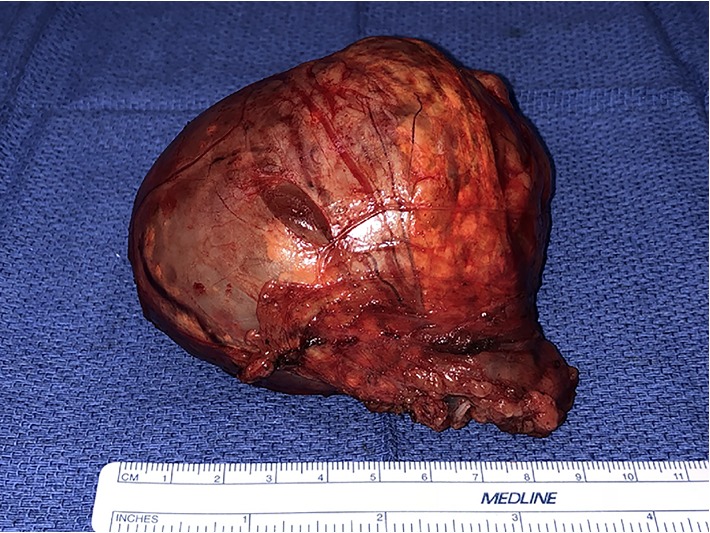
Surgically removed left adrenal tumor following robot-assisted laparoscopic adrenalectomy.

**Figure 4:**
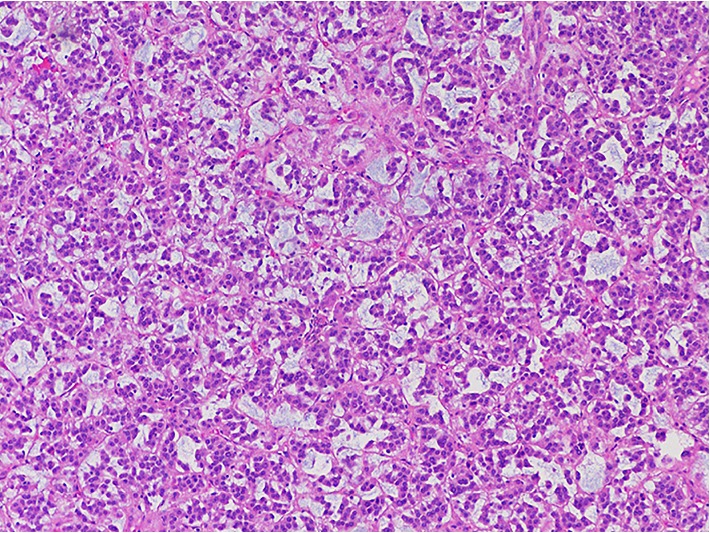
Adrenocortical carcinoma with myxoid background (H&E section, 100x)

The patient underwent robot-assisted laparoscopic left adrenalectomy (Figures 2 and 3) after appropriate alpha blockade with phenoxybenzamine and beta blockade with propranolol. Three 8 mm robotic laparoscopic ports and one 12 mm assistant port were used, two along the midline and two in the left lower quadrant. A “T-incision” was made in the posterior peritoneum allowing inferomedial reflection of the splenic flexure. The adrenal vein was identified early, ligated with polymer locking clips then divided, after which the body was dissected away from the adrenal gland. The plane of dissection was taken outside of Gerota's fascia on all aspects except in the plane adjacent to the kidney, where perinephric fat was resected *en bloc* with the adrenal mass to ensure complete resection. As is our standard practice with all extirpative surgeries, the specimen was extracted intact using an impermeable laparoscopic bag through an extended port-site. The adrenal tumor measured 9.0 × 8.0 × 7.5 cm (260 grams). Histological sections showed a cortical neoplasm with prominent myxoid changes (Figure 4). Rare mitotic activity was identified (1/50 high-power field). No cytologic atypia or atypical mitoses were seen. Capsular invasion was present (entire capsule submitted for histological examination). Histologic diagnosis was reviewed and confirmed by an expert adrenal pathologist at a tertiary cancer center with experience in tumors of the endocrine systems.

## 6. Follow Up and Outcomes

Post-operatively, the patient developed dyspnea attributed to phenoxybenzamine and propranolol, and for which work up for myocardial and thrombotic causes were negative. She was weaned off her antihypertensive medications, and the dyspnea resolved. At one month follow up, her hypertension was resolved and she no longer required antihypertensive therapy. There was no evidence of recurrence on three and nine-month post-operative contrast enhanced CT imaging.

## 7. Discussion

Adrenocortical carcinoma is a rare malignancy of the adrenal cortex with an incidence of 0.7–2 cases per million per year [1]. Variants of ACC that have been identified to date include myxoid, sarcomatoid and oncocytic subtypes. At the time of this report, a PubMed search revealed that only 42 cases of myxoid adrenocortical carcinoma have been reported in the English literature [2]. Myxoid ACC is usually diagnosed in the fifth decade of life. While conventional adrenocortical carcinoma affects females more commonly than males, the myxoid variant has been found with an equal distribution among men and women.

To determine the functional status of a suspicious adrenal mass, laboratory screening is performed including basal cortisol, testosterone, estradiol, androstenedione, 17-hydroxyprogesterone, adrenocorticotropic hormone (ACTH), and urinary free cortisol level. Confirmatory testing can be performed as needed. Aldosterone and renin levels are recommended in patients with hypertension and/or hypokalemia. Our patient with myxoid ACC did not have any evidence of endocrine function on initial functional testing, however her presenting symptoms and the tumor characteristics on magnetic resonance imaging were concerning for pheochromocytoma. A biopsy was therefore not performed for fear of triggering a catecholamine crisis.

Myxoid ACC has a prognosis reported to be either similar or worse than that of conventional ACC. In one case review involving follow up of four patients with myxoid ACC, all four patients had a disease specific mortality between 11 and 69 months [3]. The Weiss scoring classification and Ki-67 classification system have been used to assist in determining ACC malignant potential and ACC prognostic value, respectively. However, myxoid variants of adrenocortical tumors are frequently malignant even in absence of mitotic activity and necrosis. In a recent report, 7 out of 14 patients with myxoid ACC died of disease even when these tumors were small and had few mitotic figures [4]. Therefore, the usual histologic criteria such as Weiss and New Helsinki cannot be used to reliably classify these tumors.

Because of the rarity of myxoid ACC there are no clear management recommendations. Treatment of conventional ACC is based on nonrandomized trials or retrospective cohort studies. Surgical resection is the only curative treatment for ACC, although the incidence of postoperative recurrence is high even in nonmetastatic disease, which is postulated to be caused by micrometastases at the time of surgery. Some retrospective studies have shown increased recurrence rates, faster time to recurrence and higher incidence of positive margins with laparoscopic adrenalectomy [2]. Robot-assisted technology for laparoscopic adrenalectomy has been used for both benign and incidentally malignant adrenal tumors for several years, however at the time of this manuscript, there has been no reported robot-assisted laparoscopic adrenalectomy for myxoid adrenocortical carcinoma in the literature to date. If a laparoscopic approach is used for large tumors and difficult dissection is encountered with concern for invasive tumor, a low threshold for conversion to open adrenalectomy should be maintained. Regardless of approach, wide resection is recommended to improve local recurrence rates. The preference of open over laparoscopic resection for large adrenal masses, in our opinion, is based on a relative paucity of data regarding laparoscopic efficacy rather than definitive evidence of inferiority of the laparoscopic approach. There have been significant advancements in laparoscopic instrumentation, techniques and surgeon experience since the early reports of laparoscopic adrenalectomy. The robotic approach provides increased dexterity than traditional pure laparoscopic adrenalectomy, albeit with a trade-off of losing haptic feedback. We ultimately decided, that the robotic approach was a feasible and safe technique in achieving oncologic success while conferring the potential benefits in post-surgical recovery.

Due to the high rates of recurrence, adjuvant chemotherapy in the form of mitotane can be considered, however the current data is limited to retrospective studies in higher risk patients. It is unclear if adjuvant mitotane therapy is beneficial in low to intermediate risk patients. The ADIUVO trial, with planned completion date in 2020, hopes to answer this question.

In general, post-operative management for conventional ACC includes surveillance CT imaging every three months for the first two years. Imaging intervals can be extended after two years and follow up is recommended for a minimum of ten years post resection [3]. If pre-operative functional studies were positive, then these should be monitored, post operatively. No specific follow up recommendations exist for myxoid ACC.

## 8. Conclusion

Myxoid adrenocortical carcinoma is a rare and aggressive entity managed with surgical resection. Though open resection for invasive adrenal cancer remains the gold standard according to most surgical associations, minimally invasive approaches are being increasingly used, whether deliberately or not [5]. We describe the first reported robot-assisted laparoscopic adrenalectomy for invasive myxoid adrenal cortical carcinoma. With further advancement in laparoscopic technology, we will likely see similar cases in the future, hopefully with excellent oncological outcomes. Regardless of approach, sound surgical principles, including wide resection and low threshold for open conversion due to difficult dissection or concern for malignancy, must be followed to ensure superior clinical outcomes for all patients.

## Abbreviation

ACC: Adrenocortical carcinoma.

## References

[1] Kerkhofs T. M., Verhoeven R. H., Van der Zwan J. M. (2013). Adrenocortical carcinoma: a population-based study on incidence and survival in the Netherlands since 1993. *European Journal of Cancer*.

[2] Miller B. S., Ammori J. B., Gauger P. G., Broome J. T., Hammer G. D., Doherty G. M. (2010). Laparoscopic resection is inappropriate in patients with known or suspected adrenocortical carcinoma. *World Journal of Surgery*.

[3] Weissferdt A., Phan A., Suster S., Moran C. A. (2013). Myxoid adrenocortical carcinoma a clinicopathologic and immunohistochemical study of 7 cases, including 1 case with lipomatous metaplasia. *American Journal of Clinical Pathology*.

[4] Papotti M., Volante M., Duregon E. (2010). Adrenocortical tumors with myxoid features: a distinct morphologic and phenotypical variant exhibiting malignant behavior. *The American Journal of Surgical Pathology*.

[5] Ball M. W., Hemal A. K., Allaf M. E. (2017). International consultation of urological disease and european association of urology international consultation on minimally invasive surgery in urology: laparoscopic and robotic adrenalectomy. *BJU International*.

